# Experimental verification of seafloor crustal deformation observations by UAV-based GNSS-A

**DOI:** 10.1038/s41598-023-31214-6

**Published:** 2023-03-13

**Authors:** Yusuke Yokota, Masata Kaneda, Takenori Hashimoto, Shusaku Yamaura, Kenji Kouno, Yoshiaki Hirakawa

**Affiliations:** 1 Institute of Industrial Science, University of Tokyo, Tokyo, Japan; 2 Space Entertainment Laboratory, Co. Ltd., Minamisoma, Japan; 3 Yokohama National University, Yokohama, Japan

**Keywords:** Natural hazards, Geophysics, Seismology, Tectonics

## Abstract

The Global Navigation Satellite System-Acoustic ranging combination technique (GNSS-A) is the only geodetic observation method that can precisely detect absolute horizontal and vertical seafloor crustal deformations at the centimetre scale. GNSS-A has detected many geophysical phenomena and is expected to make great contributions to earthquake disaster prevention science and geodesy. However, current observation methods that use vessels and buoys suffer from high cost or poor real-time performance, which leads to low observation frequency and delays in obtaining and transmitting disaster prevention information. To overcome these problems, a new sea surface platform is needed. Here, we present an unmanned aerial vehicle (UAV) system developed for GNSS-A surveys capable of landing on the sea surface. Submetre-level seafloor positioning is achieved based on real-time single-frequency GNSS data acquired over an actual site. UAV-based GNSS-A allows high-frequency, near real-time deployment, and low-cost seafloor geodetic observations. This system could be deployed to acquire high-frequency observations with centimetre-scale accuracies when using dual-frequency GNSS.

The Global Navigation Satellite System-Acoustic ranging combination technique (GNSS-A) is a geodetic observation technology for horizontal and vertical seafloor positioning that combines GNSS and acoustic ranging on a sea surface platform, typically a vessel (Fig. 1). Since it is not possible to communicate with devices on the deep seafloor using radio waves, the sea surface platform position is determined by the GNSS and an attitude metre, and the distance from the platform to the seafloor is determined by acoustic ranging. The seafloor position can be determined by moving the platform along survey lines and taking measurements from various angles.

**Figure 1:**
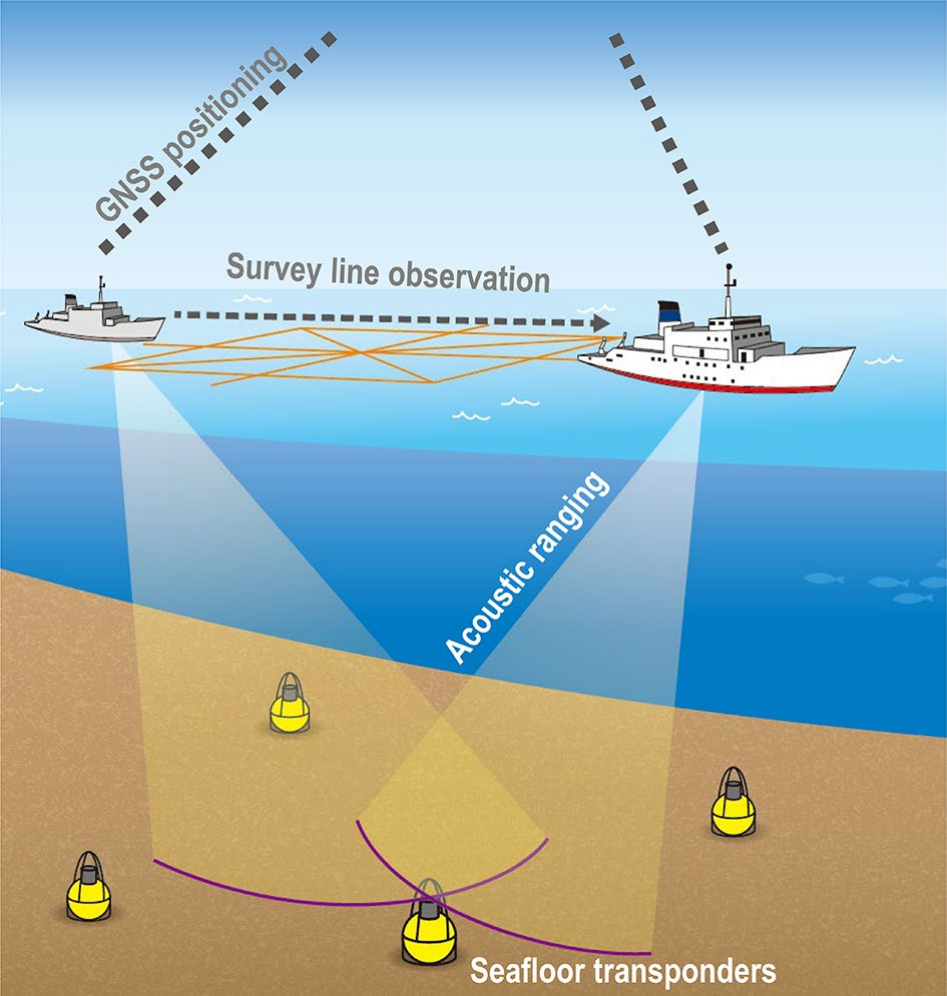
Schematic illustration of GNSS-A observation system. The GNSS observations and the acoustic ranging to the seafloor transponders are performed on the sea surface platform (vessel in this figure) moving along the survey line. This figure was modified after Refs^14, 41^.

GNSS-A, proposed in the 1980s^1^, has been deployed as a vessel-mounted system that consists of large acoustic arrays and time keeping equipment^2–6^. The geodetic absolute horizontal and vertical crustal deformations obtained using this method are important for investigating plate tectonics and earthquakes. Observations can be made simply by installing a seafloor station in advance. GNSS-A has detected large seafloor crustal deformations^7^ due to megathrust earthquakes along plate boundaries and plate boundary coupling conditions^8,9^ for understanding earthquake mechanisms and earthquake cycles. In recent years, the number of observation targets that require high temporal resolution, such as slow slip events^10^ and temporal changes following postseismic deformations^11,12^, has increased.

Since the 1990s, land based GNSS has been used to detect crustal deformation phenomena (at high temporal resolutions) that accompany earthquakes and various temporal changes^13^. Current vessel-based GNSS-A systems have sufficient accuracy to detect long-term fluctuations in seafloor crustal deformations, but the observation frequency is insufficient^14^. Large vessels have significant restrictions in terms of cost (e.g., energy cost alone is more than $1000/day) and mobility (approximately 20–30 km/h). Absolute crustal deformation immediately after a disaster, which is necessary for estimating the severity of a disaster, can be detected by land-based GNSS^15,16^. However, GNSS-A cannot be used immediately after an event due to various vessel limitations^7^. The current GNSS-A technique thus has limitations in terms of observation frequency and speed of observation (Table 1).

**Table 1:** Comparison of sea surface platforms capable of GNSS-A observations in terms of energy cost, real-time operation ability, and sea surface control performance versus ocean currents.

	Vessel	Buoy	UAV
Energy cost	More than $1000/day	Zero cost after installation	Less than $100/day
Real-time operation	Cannot be immediately deployed	Cannot move between sites (self-propelled buoys have similar capabilities to those of a vessel)	Can travel at more than approximately 80 km/hour
Control performance versus ocean currents	Can move without problems	Cannot move (self-propelled buoys cannot move in areas with strong currents such as Kuroshio region)	Can move without problems

Experiments have been conducted in which GNSS-A equipment was mounted on mooring and self-propelled buoys and also a wave glider^17–21^. Buoy-based GNSS-A, described in Table 1, has poor sea surface control performance, and the buoy position is strongly influenced by the current. For example, in the Kuroshio region (strong tidal current near the Japan Islands), the sea surface control performance is insufficient; thus, it may be impossible to reach the observation site. In addition, such poor control performance prevents observation immediately after an earthquake.

Unmanned aerial vehicles (UAVs) are utilized in various fields, including geophysics^22,23^. UAVs are broadly categorized into small helicopter and large aircraft types. The former has short-distance flight capability, high mobility, and small payload capacity, and the latter has long-distance flight capability, low mobility, and large payload capacity. The scientific applications of UAVs are rapidly increasing^24–28^. In marine engineering, data collection using a helicopter-type UAV has been proposed^29,30^, and the development of actual ocean observations has begun^31–33^.

Figure 2A shows near-sea observation applications that have already been implemented for meteorological, volcanic and oceanographic sciences. Many of the tasks that require landing on the sea surface in distant oceanic regions, such as those shown in Fig. 2B, have not yet been realized with UAVs. Helicopters cannot be used to autonomously conduct seafloor geodetic observations (target depth: 1000 m or more) because of their flight distance limitations. To achieve these observations with UAVs, a float plane UAV is needed.

**Figure 2:**
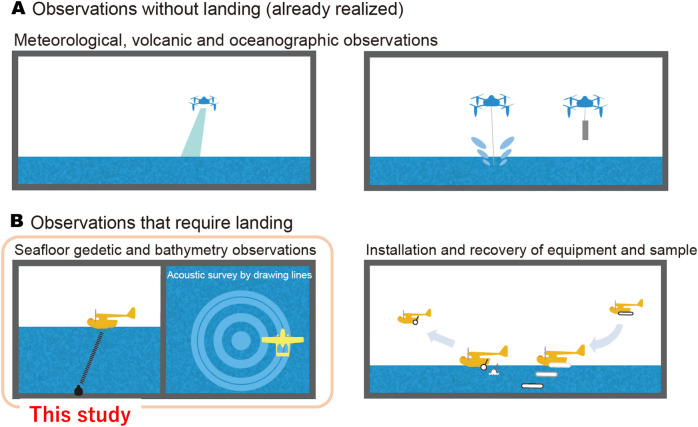
Schematic of UAV ocean observations. (**A**) Observations without landing (already in operation). (**B**) Observations that require landing. Seafloor geodetic and bathymetry observations and the installation and recovery of equipment and samples require sea surface landing technology. In this study, seafloor geodetic observation equipment was installed on a UAV.

In this research, an observation experiment was carried out using GNSS-A equipment installed on a float plane UAV HAMADORI6000 prototype model, which was recently developed by Space Entertainment Laboratory Co., Ltd. (Fig. 3A and Movie S1). It was designed for taking off and landing on the sea surface. This kind of UAV is much less expensive than vessels in terms of manufacturing and fuel costs (Table 1). It was designed to fly at a speed of 80 km/h or more, allowing it to quickly reach an observation site. Because it can also move at high speed on the sea surface, it is capable of acquiring observations even in a strong-current environment.

**Figure 3:**
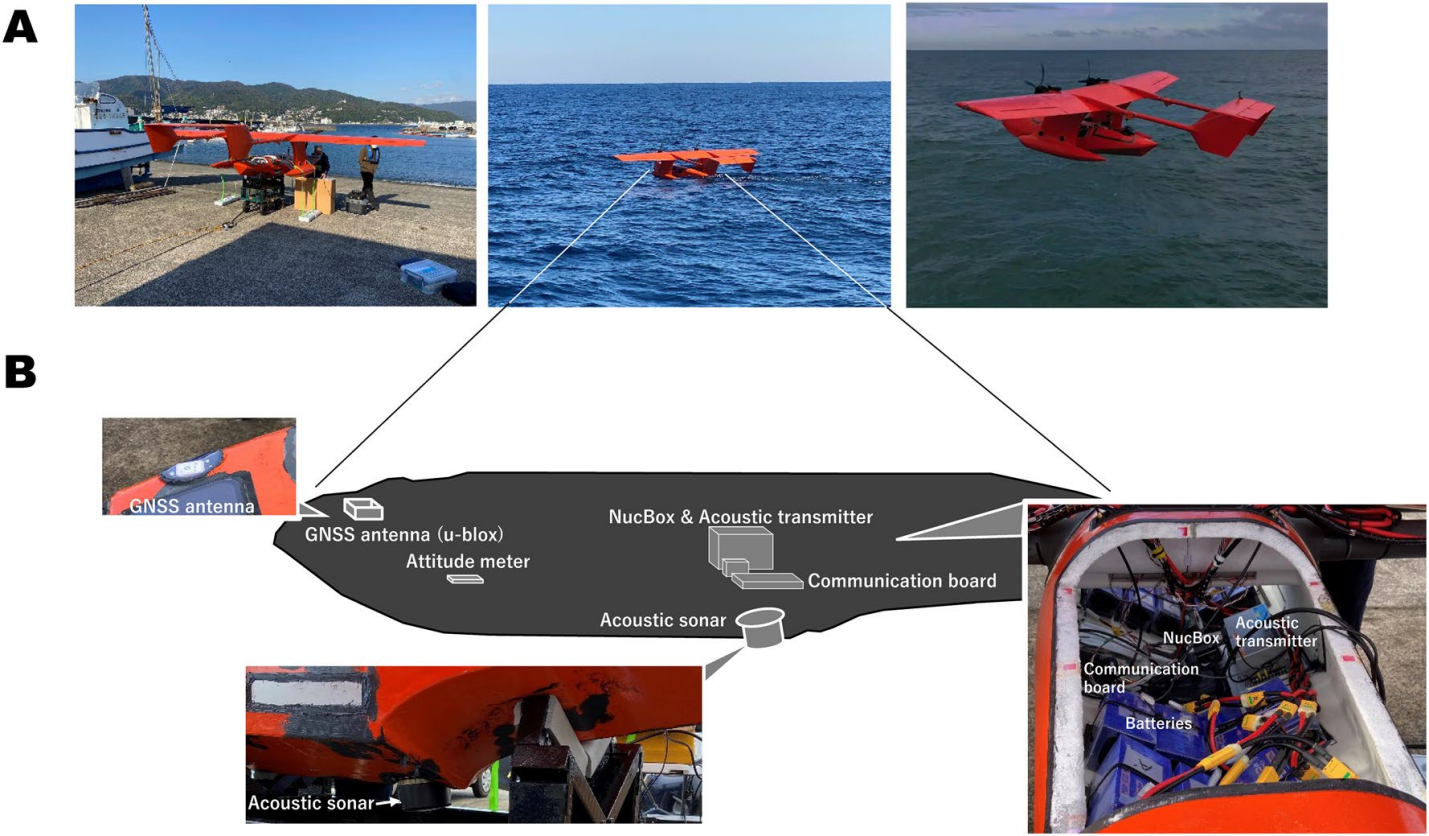
(**A**) Photographs of the UAV HAMADORI6000 prototype model used in this study. From left to right: before departure, during observation, and during flight test. (**B**) Internal configuration with photographs of the GNSS antenna, acoustic sonar, and control system.

The payload weight for the UAV should be lower than that for vessels and buoys to allow longer flights. This UAV was equipped with the specially developed lightweight and compact observation equipment (Fig. 3B). Movie S2 shows the movement of the UAV along the survey line during observations. Observations can be stably performed while the acoustic sonar is submerged in seawater. During observations, flight, sea surface navigation, and sound transmission are remotely controlled by a flight controller (Holybro Pixhawk 4). Since this experiment was preliminary, the position of the seafloor was determined with metre-level-precision, which was accomplished using only real-time data from the UAV control system (dual-frequency GNSS was not used).

## Results

### Observation survey

**Figure 4:**
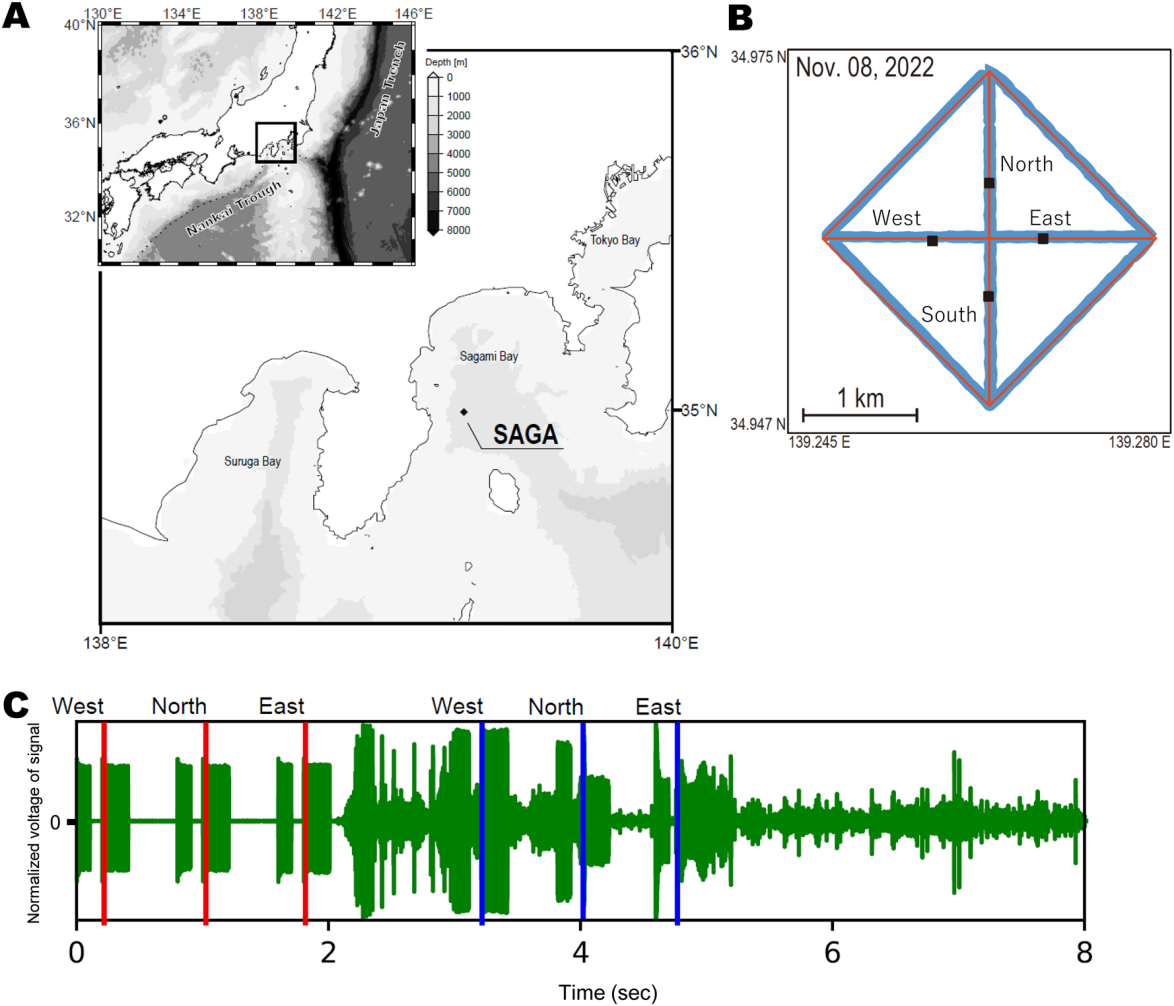
(**A**) Location of the SAGA site. (**B**) Comparison between the planned survey line and actual observation track on November 8, 2022. Red and blue lines indicate the planned and observation tracks, respectively. Black squares indicate the locations of the four seafloor stations. (**C**) Example of an actual signal. The two-way travel time was measured from the time difference between the transmitted signal (red line) and the received signal (blue line). This is a case of continuous transmission for west, north, and east stations from near the centre of the observation site. The vertical axis is the voltage, which has been normalized by adjusting the gain of the physical oscillation of the sonar. Since acoustic sonar applies Auto Gain Control (AGC) during reception, the actual voltage value is unknown.

Figure 4A shows the location of the SAGA site (latitude: 34.962 N, longitude: 139.263 E, depth: approximately 1350 m) within the Seafloor Geodetic Observation Array (SGO-A) operated by the Japan Coast Guard. Four seafloor stations are arranged on a circle approximately 500 m from the centre. The UAV-based GNSS-A observations were conducted on November 8, 2022. The weather and sea surface conditions on the observation days were fine, with a maximum wind speed of approximately 8 m/s and a maximum wave height of approximately 1.5 m. The UAV navigated automatically to follow multiple waypoints on the planned survey line (Fig. 4B). The UAV moved along the survey line at a speed of approximately 2 knots. For many seafloor acoustic observations, measurements within 100–200 m from the survey line are sufficient and this UAV has this capability. The survey took approximately 3 h and consumed 5703 Wh of energy. To measure more distant observation points, a large energy source is required for flight. We plan to use a gasoline engine for this task. The energy cost for UAV-based observation is negligible compared to that for vessel-based observation.

### Signal

In GNSS-A, underwater ranging is often performed using acoustic signals based on the M-sequence. In this study, the ninth-order M-sequence signal, where one digit was represented by four waves at 10 kHz (total length: 204.8 ms), was used for acoustic ranging, as done in the SGO-A observations^14^. Figure 4C shows an example of a signal after a round trip to the seafloor stations.

In vessel-based GNSS-A, sonar system is deployed several metres below the sea surface, so the obstructive effect that bubbles generated near the sea surface have on acoustic communication can be ignored. In UAV-based GNSS-A, the acoustic sonar was arranged as shown in Fig. 3B and was not far from the sea surface. However, this UAV is relatively large; therefore, the incidence of such cases was low. In this experiment, the data acquisition rate was approximately 90%. As a result, there were almost no communication interruptions caused by the effect of bubbles in this experiment.

### Dataset

This observation experiment evaluated real-time data acquisition, observation accuracy, and communication. The dataset used for determining the seafloor position was real-time data from the real-time position information determined by a u-blox Neo-M8N GPS/GLONASS receiver in the UAV body (observation accuracy of approximately 1 m or less). The acquired data were in a data format that conformed to the GARPOS format^34^ and are published as open data^35^. Since no sound speed profile (SSP) was obtained during this experiment or within the observations acquired by the buoy-based GNSS-A, we used the SSP estimated from past SSP information for analysis since SGO-A had previously accumulated sufficient observation data. For sites for which no observations were performed, it was necessary to estimate the SSP from previous observations.

### Station position results

**Figure 5:**
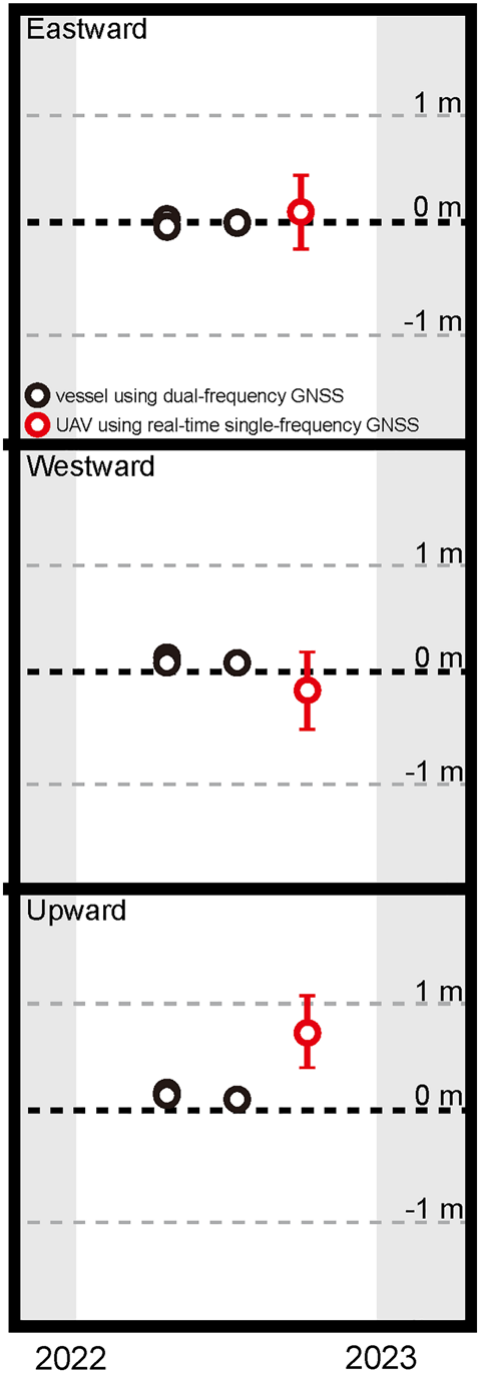
Obtained seafloor position time series. Eastwards, northwards, and upwards components are shown in order from the top. Black and red circles indicate results obtained by vessel-based GNSS-A using dual-frequency GNSS and UAV-based GNSS-A using single-frequency GNSS, respectively. Position error bars indicate a width of ± 1 σ obtained in GARPOS.

The observed data were analysed using the software GARPOS version 1.0.1^34,36^. See the “Methods” section for details about the observation and analysis methods. The obtained seafloor station positions are shown in Fig. 5; they are compared with the seafloor positions estimated from the vessel-based GNSS-A data^37,38^. No significant crustal movement event occurred during this period. The value of each component of UAV-based GNSS-A agrees with that of vessel-based GNSS-A, suggesting that the actual crustal deformation field was correctly measured, although the up and down motion was affected by the vertical accuracy of real-time single-frequency GNSS positioning.

The accuracy of the horizontal positions obtained from the vessel-based GNSS-A data is roughly within ± 2–4 cm (the highest observation accuracy of the current GNSS-A^14^). On the other hand, the horizontal positioning variation obtained from the UAV-based GNSS-A's real-time data is generally within approximately ± 35 cm since we used the real-time single-frequency GNSS positioning values. The fact that the vertical component was biased by approximately 70 cm was due to using single-frequency GNSS. Accuracy using dual-frequency GNSS will be investigated in the future. This loss of positioning variation can be explained by differences in GNSS and cannot be attributed to the design of the UAV observation system or the equipment used. In the future, centimetre-level accuracy can be achieved by using dual-frequency GNSS and setting the appropriate parameters in the analysis.

The values determined here can be analysed immediately after the observations are stored within the UAV body. Therefore, the UAV-based GNSS-A can observe the seafloor position with the above accuracy virtually in real time. The real-time data can be used for detecting crustal deformations of approximately 1 m or more. For example, the amount of crustal deformation that occurs immediately after an earthquake of approximately magnitude 7 or more can be detected. The real-time UAV-based GNSS-A observation data can contribute to the prediction of surrounding seismic activity by acquiring the horizontal seafloor movement immediately after an earthquake and detecting early postseismic deformation. A UAV equipped with dual-frequency GNSS can acquire high-frequency observations of slow temporal changes in seafloor crustal deformations, such as those caused by a slow slip or postseismic events.

## Discussion

It was experimentally demonstrated that UAV-based GNSS-A can perform real-time continuous observations at low cost. The approach presented in this paper is to transfer GNSS-A equipment from vessels to UAVs. This could be achieved thanks to the fact that error models and correction methods have already been established to some extent through pioneering research on vessel-based GNSS-A surveys (e.g., acoustic modelling of subsurface structures, assessing conditions regarding the placement of seafloor stations, refining methods of collecting acoustic signals)^14,34,37^. During actual operation of UAV-based GNSS-A survey systems, it is essential to properly operate these technologies.

To realize UAV-based GNSS-A observations with centimetre-level accuracy, a dual-frequency GNSS antenna is needed. Consideration must be given to GNSS antenna placement to prevent UAV movements on the sea surface from interfering with GNSS positioning. For a wing-shaped UAV, the antenna must be installed near the wing so as not to be obstructed by the wing and other parts. Recently, we have started developing this type of UAV that can appropriately accommodate dual-frequency GNSS antennas, and we believe that it will be possible to determine the final cm-level seafloor position during the next observation experiment.

Compared to vessels, UAVs are similar to buoys and experience considerable movement at sea level. From the results of this experiment, we could not detect any problems with the acoustic communication and analysis results due to this effect, but in the future, we plan to conduct engineering experiments such as tank tests and sea-based tests to clarify the observation limit. The effects of reflections and sound disturbances and superimpositions by the aircraft frame should also be investigated in the future.

Direct ocean and seafloor observations using a float plane UAV are expected to be useful in various marine engineering fields. The transportation of marine robots (such as autonomous underwater vehicles and remotely operated vehicles) within the sea to serve as a base of communication with the sea surface will be important for marine robotics technology. Undersea exploration and observations using undersea acoustic sonar can be applied to most marine research fields. This kind of UAV can also be applied to water sampling for seafloor volcano surveys and biological surveys. This technology is suitable for repeat observations or observations of sea regions that are dangerous or expensive for people to get to and is expected to improve observation speed and frequency, reduce cost, and increase sustainability. Surface UAVs could join vessels and buoys as a sea surface platform.

## Methods

The UAV used in this study was a float plane UAV HAMADORI6000 prototype model (Fig. 3A) recently developed by Space Entertainment Laboratory Co., Ltd. It has a wingspan of 6 m and a cruising flight speed of approximately 80 km/h or more. The take-off distance is approximately 30 m. The UAV can freely take off and land on open seas (Movie S1). As a product model, it is designed to be capable of flying 750 km for up to 8 h.

The GNSS-A equipment (Fig. 3B) was a lighter version than the equipment described in Ref.^14^. The GNSS equipment was a u-blox Neo-M8N GNSS receiver and an ANT-2B antenna. The attitude metre and flight controller were an inertial measurement unit (ICM-20689, TDK InvenSense) and a Holybro Pixhawk 4, respectively. The acoustic sonar was a small cylindrical transducer (ITC-3013, Gavial ITC), and the processing PC was a NucBox (GMKtec) with an Intel Celeron J4125 processor. The total weight of the observation equipment payload was approximately 6 kg, which is significantly lighter than general GNSS-A equipment.

Acquired data^35^ were analysed using the GARPOS version 1.0.1 software ^34,36^. The seafloor positions were analysed based on the assumption that the shape of the four seafloor stations that comprise the array is invariant based on Ref.^39^. In this analysis, the following *Γ* (defined in Ref.^34^ based on Ref.^40^) function was estimated as a model value of the ocean field:

1 $$\Gamma \, = \,\alpha_{0} \, + \,\alpha_{1} \cdot P\, + \,\alpha_{2} \cdot X,$$

where ***α***_0_,*** α***_1_, and*** α***_2_ are parameters related to the entire SSP and the fluctuation of the sound speed field acquired from the sea surface station and seafloor station, respectively. ***P*** and ***X*** are the standardized positions of the sea surface and seafloor stations, respectively. The time series of these parameters were estimated simultaneously with the seafloor position. The number of knots in the estimated time series was set to 15, as in Ref.^34^. The hyperparameters were the same as in Ref.^34^. However, considering the positioning accuracy determined by real-time GNSS, the hyperparameter that determines the smoothness in the time direction (*μ*_T_) was set to 2 min.

## Supplementary Information

Movie S1. UAV taking off, flying and landing on the sea surface. It was filmed on different days by an observation drone.

Movie S2. UAV performing observations on the sea surface. While the UAV navigates a preplanned survey line on the sea surface, it performs acoustic communication with a station installed at a depth of 1350 m.

## Data Availability

The datasets generated and analysed during the current study are available in the Zenodo repository, 10.5281/zenodo.7471959^35^. All codes and data used in the current study are available at 10.5281/zenodo.6414642^36^ and https://www1.kaiho.mlit.go.jp/KOHO/chikaku/kaitei/sgs/datalist_e.html^38^.
